# Epithelial-Mesenchymal Transition in Asthma Airway Remodeling Is Regulated by the IL-33/CD146 Axis

**DOI:** 10.3389/fimmu.2020.01598

**Published:** 2020-07-22

**Authors:** Zhixiao Sun, Ningfei Ji, Qiyun Ma, Ranran Zhu, Zhongqi Chen, Zhengxia Wang, Yan Qian, Chaojie Wu, Fan Hu, Mao Huang, Mingshun Zhang

**Affiliations:** ^1^Department of Respiratory and Critical Care Medicine, The First Affiliated Hospital of Nanjing Medical University, Nanjing, China; ^2^State Key Laboratory of Reproductive Medicine, Nanjing Medical University, Nanjing, China; ^3^NHC Key Laboratory of Antibody Technique, Department of Immunology, Nanjing Medical University, Nanjing, China

**Keywords:** epithelial-mesenchymal transition, IL-33, CD146, asthma, allergy

## Abstract

Epithelial-mesenchymal transition (EMT) is essential in asthma airway remodeling. IL-33 from epithelial cells is involved in pulmonary fibrosis. CD146 has been extensively explored in cancer-associated EMT. Whether IL-33 regulates CD146 in the EMT process associated with asthma airway remodeling is still largely unknown. We hypothesized that EMT in airway remodeling was regulated by the IL-33/CD146 axis. House dust mite (HDM) extract increased the expression of IL-33 and CD146 in epithelial cells. Increased expression of CD146 in HDM-treated epithelial cells could be blocked with an ST2-neutralizing antibody. Moreover, HDM-induced EMT was dependent on the CD146 and TGF-β/SMAD-3 signaling pathways. IL-33 deficiency decreased CD146 expression and alleviated asthma severity. Similarly, CD146 deficiency mitigated EMT and airway remodeling in a murine model of chronic allergic airway inflammation. Furthermore, CD146 expression was significantly elevated in asthma patients. We concluded that IL-33 from HDM extract-treated alveolar epithelial cells stimulated CD146 expression, promoting EMT in airway remodeling in chronic allergic inflammation.

## Introduction

Asthma is a disease that is characterized by airway inflammation, airway remodeling, and airway hyperresponsiveness (1). Airway remodeling is described as a change in the composition, thickness or volume of airway walls, including subepithelial fibrosis, and increased smooth muscle composition, in asthmatic patients compared to normal individuals (2). Epithelial-mesenchymal transition (EMT) is a pathophysiological process induced by multiple signaling pathways centered on TGF-β and refers to the loss of function of epithelial cells and their transformation to mesenchymal cells, including a decrease in E-cadherin, and an increase in N-cadherin expression (3–5). An increasing number of studies have demonstrated that increased EMT plays an important role in airway remodeling in asthma (5, 6).

CD146 was originally acknowledged as a tumor marker for melanoma (MCAM). As a multifunctional molecule (7), CD146 plays diverse biological roles in tumors, atherosclerosis, systemic sclerosis, and other diseases (8–10). CD146 in macrophages promotes cell adhesion and foam cell formation (8). CD146 in CD4^+^ T cells is associated with Th17 differentiation in systemic sclerosis (9). CD146 is also associated with pulmonary infections, in which it promotes the adherence of bacteria or viruses to airway epithelial cells (11–14). Increased expression ofCD146 in gastric cancer leads to decreased expression of E-cadherin and increased expression of N-catenin and vimentin (15). CD146 also regulates the EMT process in hepatocellular carcinoma via the MAPK1 signaling pathway, which exacerbates the invasion and metastasis of hepatocellular carcinoma (16). These studies suggest that the EMT process is associated with cancer progression. Increased expression of CD146 in the airway epithelial cells of asthma patients was recently discovered, and IL-13 (a type 2 inflammatory cytokine) regulates the expression and function of CD146 in airway epithelial cells (11, 12). Although the regulation of EMT by CD146 has been extensively reported in studies of tumor metastasis (17), the roles of CD146 in asthma EMT and airway remodeling have not been explored.

Interleukin-33 (IL-33) is a member of the IL-1 cytokine family and is expressed in fibroblasts, endothelial cells, epithelial cells, and other cell types (18, 19). Once bound with the membrane receptor ST2, IL-33 activates the MyD88/NF-κB signaling pathway (20) and induces the type 2 response in CD4^+^ T cells, which release IL-4, IL-5, and IL-13 (19). Serum IL-33 and the soluble form of ST2 are closely associated with asthma disease progression (21) and exacerbation (22). Moreover, recent studies have shown that IL-33 is involved in asthma airway collagen deposition, suggesting that IL-33 may be involved in the EMT process in the lung (23–25). The regulation of IL-33 signaling related to CD146 expression and the EMT process in asthma, however, remains largely elusive. In the present study, we demonstrated that IL-33 increased the expression of CD146, which promoted the EMT process in asthma.

## Materials and Methods

### Animals and a Murine Model of Asthma

Specific pathogen-free (SPF) female C57BL/J mice aged 6–8 weeks were obtained from the Laboratory Animal Center, Nanjing Medical University (Nanjing, China). CD146 knockout (KO) mice on a C57BL/J background were obtained from Cyagen, Suzhou, China. IL-33 KO mice on a C57BL/J background were obtained from Dr. Hong Zhou (Department of Immunology, Nanjing Medical University). All animal treatments were approved by the Nanjing Medical University Ethics Committee (IACUC 1709011).

To establish a murine model of asthma, the mice were intranasally administered house dust mite (HDM, Greer Laboratories, Lenoir, NC, USA) extract (25 μg of HDM extract dissolved in 40 μL of phosphate-buffered saline) 5 days/week for 5 weeks. All mice were treated with HDM extract under isoflurane anesthesia and were ultimately sacrificed (26).

### Cell Culture

The mouse pulmonary epithelial cell lines MLE-12 and A549 were obtained from ATCC (VA, USA) and cultured in DMEM containing 10% fetal bovine serum (FBS), 100 IU/ml penicillin and 100 μg/ml streptomycin in a 5% CO_2_ atmosphere at 37°C. MLE-12 or A549 cells were seeded in 6-well plates or 24-well plates overnight and then treated with HDM extract or the cytokine IL-33 for the indicated durations. Primary alveolar epithelial cells from mice were purified using 0.1% collagenase, 0.25% trypsin, and DNase I and were selected with mouse IgG (36111ES60, Yeasen, China) as previously described (27). To exclude the potential effects of lipopolysaccharide (LPS) contamination, HDM extract was treated with the ToxinEraser^TM^ endotoxin removal Kit (L00338, Genscript, China). The purified product was the major constituent of HDM.

### Cell Transfection

MLE-12 cells were seeded and incubated overnight before transfection. The CD146 expression plasmid, an siRNA plasmid, or blank vehicles (Abmgood, China) were mixed with Lipofectamine 2000 (Invitrogen, USA) in DMEM without FBS, penicillin or streptomycin for 25 min and were then transfected into MLE-12 cells at 60–80% density in DMEM for 48 h. The cells were treated with HDM extract or PBS for 24 h before total protein extraction.

### Western Blotting

Total protein from the cells or tissues was lysed with RIPA buffer (89900, Thermo, USA) containing protease and phosphatase inhibitors (78443, Thermo, USA) on ice for 20 min. Then, the samples were centrifuged for 10 min, and the supernatants were collected and transferred into new EP tubes. The protein concentrations were measured by a BCA assay (P0012S, Beyotime, China). The proteins were separated by SDS-PAGE and transferred to polyvinylidene difluoride (PVDF) membranes at 300 mA. The PVDF membranes were blocked with 5% skim milk powder for 1 h at room temperature and were then incubated with primary antibodies (Table 1) at 4°C overnight. The PVDF membranes were washed with TBST 4 times for 5 min each and were then incubated with goat anti-rabbit HRP IgG (EarthOx Life Sciences) or goat anti-mouse HRP IgG (EarthOx Life Sciences) for 1 h at room temperature. The PVDF membranes were washed with TBST 4 times for 7 min each. The specific antibody-bound proteins were visualized with the Immobilon Western Chemiluminescent HRP Substrate (Millipore, MA, USA) and the G:Box gel doc system (Syngene, UK).

**Table 1:** Antibodies in the study.

**Antibody**	**Brand name**	**Product code**	**Source**	**Dilutability**
Anti-CD146 antibody	Abcam	ab75769	Cambridge, UK	1:1000
Anti-IL-33 antibody	Abcam	ab54385	Cambridge, UK	1:1000
Anti-SPD antibody	Abcam	ab220422	Cambridge, UK	1:1000
Anti- E-cadherin antibody	Abcam	ab76055	Cambridge, UK	1:1000
Anti-N-cadherin antibody	Abcam	ab76011	Cambridge, UK	1:5000
Anti-α-SMA antibody	Abcam	ab7817	Cambridge, UK	1:200
AntiTGF-β antibody	Abcam	ab170874	Cambridge, UK	1:1000
Anti- fibronectin antibody	Proteintech	15613-1-AP	Wuhan, Hubei, China	1:1000
Anti-beta-actin antibody	Cell signaling technology	#4970	Beverly, MA	1:1000
Anti-P38 antibody	Cell signaling technology	#8690	Beverly, MA	1:1000
Anti-P38 (phospho-Thr180/Tyr182) antibody	Cell signaling technology	#4511	Beverly, MA	1:1000
Anti-P44/42 antibody	Cell signaling technology	#4695	Beverly, MA	1:1000
Anti- P44/42(phospho-Thr202/Tyr204) antibody	Cell signaling technology	#4370	Beverly, MA	1:1000
Anti-P65 antibody	Cell signaling technology	#8242	Beverly, MA	1:1000
Anti- P65 (phospho-Ser536) antibody	Cell signaling technology	#3033	Beverly, MA	1:1000
Anti-JNK antibody	Cell signaling technology	#9252	Beverly, MA	1:1000
Anti-JNK(phospho-Thr183/Tyr185) antibody	Cell signaling technology	#4668	Beverly, MA	1:1000
Anti-STAT3 antibody	Cell signaling technology	#4904	Beverly, MA	1:1000
Anti-STAT3 (phospho-Tyr705) antibody	Cell signaling technology	#9145	Beverly, MA	1:1000
Anti-SMAD3 antibody	Cell signaling technology	#9523	Beverly, MA	1:1000
Anti-SMAD3 (phospho-Ser423/425) antibody	Cell signaling technology	#9520	Beverly, MA	1:1000

### Quantitative Real-Time PCR

Total RNA was extracted from cells using the TaKaRa Universal Total RNA Extraction Kit (Dalian, China) and was then used to synthesize cDNA using PrimeScript RT master mix (TaKaRa). The expression of specific RNAs was quantified by using SYBR Green Universal PCR master mix (TaKaRa) in a StepOnePlus Real-Time PCR System (ABI, USA). The primer sequences used for real-time PCR were synthesized by Genescript. The primer sequences are as follows: CD146 forward, 5′- GGACCTTGAGTTTGAGTGG−3′; CD146 reverse, 5′- CAGTGGTTTGGCTGGAGT−3′; β-actin forward, 5′- GAGAAGCTGTGCTATGTTGCT−3′; and β-actin reverse, 5′- CTCCAGGGAGGAAGAGGATG -3'.

### Immunofluorescence

After treatment with HDM extract or PBS for 24 h, the culture medium was removed, and the MLE-12 cells were washed in PBS 3 times. The cells were then fixed in 4% paraformaldehyde at 4°C for 15 min, followed by 3 washes with PBS. Afterwards, the cells were blocked with 5% goat serum for 1 h at room temperature and were incubated with mouse anti-E-cadherin, rabbit anti- N-cadherin or rabbit anti-SPD primary antibody at 4°C overnight. The cells were washed with PBS 3 times and incubated with Alexa Fluor 555 donkey anti-mouse IgG (H+ L) or Alexa Fluor 647 donkey anti-rabbit IgG (H+L) at 37°C for 1 h in the dark. Next, the cells were washed with PBS and stained with DAPI (4′,6-diamidino-2-phenylindole; Yeasen, China) at 37°C for 10 min in the dark. Images were visualized with a ZEISS LSM710 confocal fluorescence microscope or an Olympus IX73 fluorescence microscope.

### Airway Responsiveness

The FinePointe RC System (Buxco Research Systems, Wilmington, NC) was used to measured airway responsiveness. Mice were challenged with aerosolized PBS and methacholine to measure lung resistance. The airway resistance values were recorded for 3 min after each challenge. Then, we calculated the average airway resistance (28).

### Differential Counts of Inflammatory Cells in BALF

The bronchoalveolar lavage fluid (BALF) was collected from mice and centrifuged to separate the supernatant and sediment. The sediment was resuspended in PBS and measured with a blood cell analyzer (ADVIA 2120i).

### Histological Staining

Lung tissues were fixed in 4% paraformaldehyde and embedded in paraffin. Tissue sections were stained with H&E, PAS, and Sirius red. Images were visualized with a Zeiss Axio Examiner microscope.

### Immunohistochemistry

Lung tissues were fixed in 4% paraformaldehyde and embedded in paraffin. Tissue sections were blocked with 5% goat serum for 30 min at 37°C and incubated with mouse anti-E-cadherin at 4°C overnight. Tissue sections were then incubated with horseradish peroxidase-conjugated secondary antibodies for 1 h at room temperature. 3,3-Diaminobenzidine (DAB) was used as a color developer, and hematoxylin was used for counterstaining. Images were visualized with a Zeiss Axio Examiner microscope.

### ELISA

Mouse blood was collected and centrifuged to extract the serum. Total IgE was measured with an ELISA kit (432401, Biolegend, USA). The lungs of mice were ground and centrifuged to extract the supernatant. The cytokines IL-4 (431104, Biolegend, USA), IL-5 (431204, Biolegend, USA), IL-13 (900-K207, PeproTech, USA), IL-33 (88-7333-88, Invitrogen, USA) and IFN-γ (430804, Biolegend, USA) in the supernatants of lung homogenates were measured using an ELISA kit. Collagen I in the lung homogenates was measured using an ELISA kit (E-EL-M0325c, Elabscience, China). Soluble CD146 in human plasma (E-EL-H2403c, Elabscience, China) was measured using commercial ELISA kits. All ELISA experiments were performed according to the instructions provided by the manufacturers.

### Statistical Analysis

Statistical analysis was performed using GraphPad Prism 5 (La Jolla, CA), and the data are displayed as the means ± SEM. Images from the Western blotting or immunofluorescence results were analyzed with ImageJ. Student's *t*-test or one-way ANOVA was applied to assess the statistical significance. A value of *P* < 0.05 was considered statistically significant (^*^P < 0.05; ^**^P < 0.01; ^***^P < 0.001; and #*P* > 0.1).

## Results

### HDM Promoted CD146 Expression in Alveolar Epithelial Cells via IL-33/ST2 Signaling

**Figure 1:**
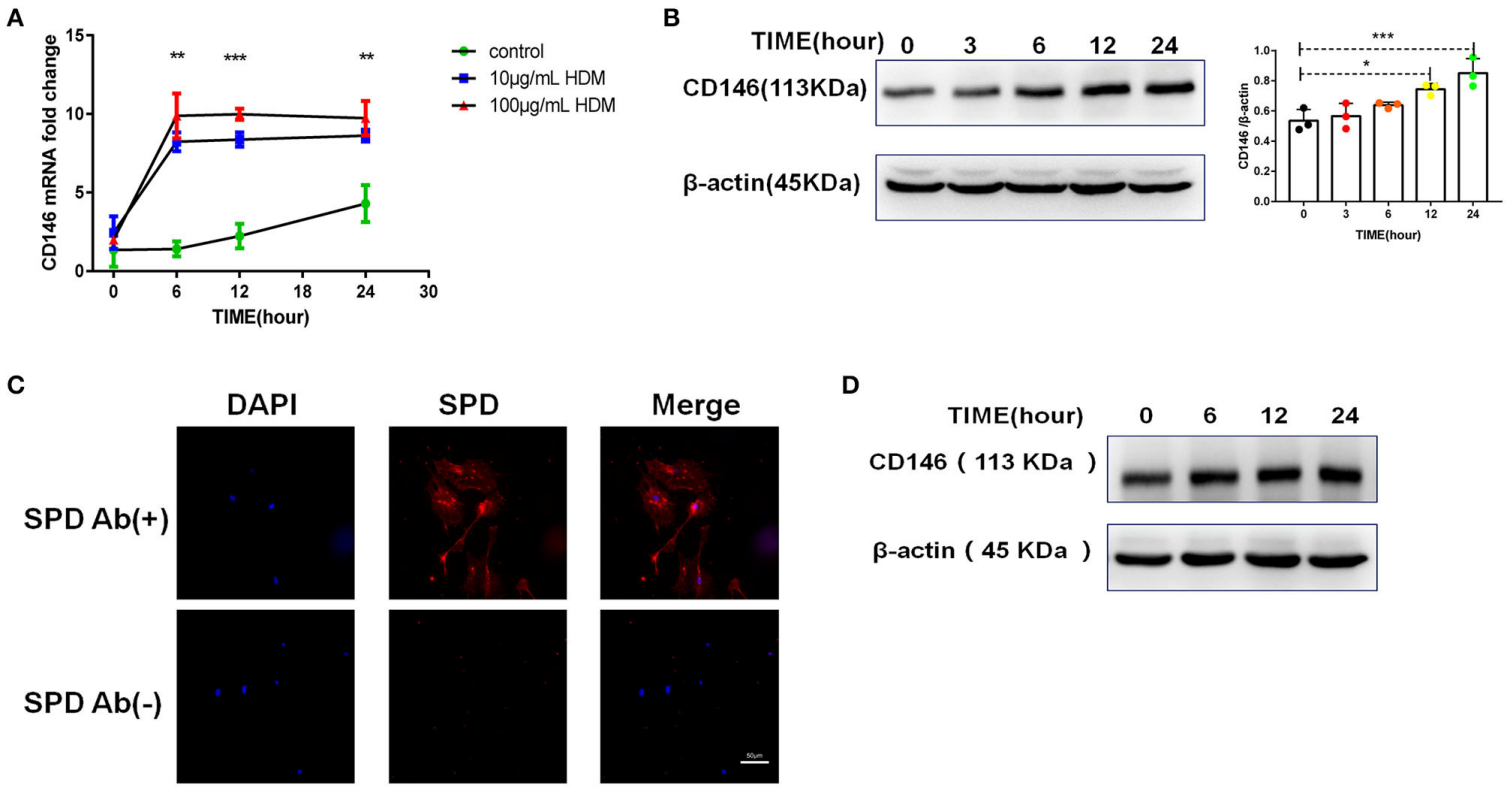
HDM promoted CD146 expression in alveolar epithelial cells. **(A)** CD146 mRNA in HDM-treated MLE-12 cells was measured by qRT-PCR. **(B)** Western blot analysis of CD146 expression in MLE-12 cells treated with HDM extract (100 μg/ml). **(C)** The level of SPD in primary alveolar epithelial cells was measured by immunofluorescence. Ab(+): stained with anti-SPD antibody; Ab(–): stained with isotype antibody **(D)** Western blot analysis of CD146 expression in primary alveolar epithelial cells treated with HDM extract (100 μg/ml). *P < 0.05; **P < 0.01;***P < 0.001.

**Figure 2:**
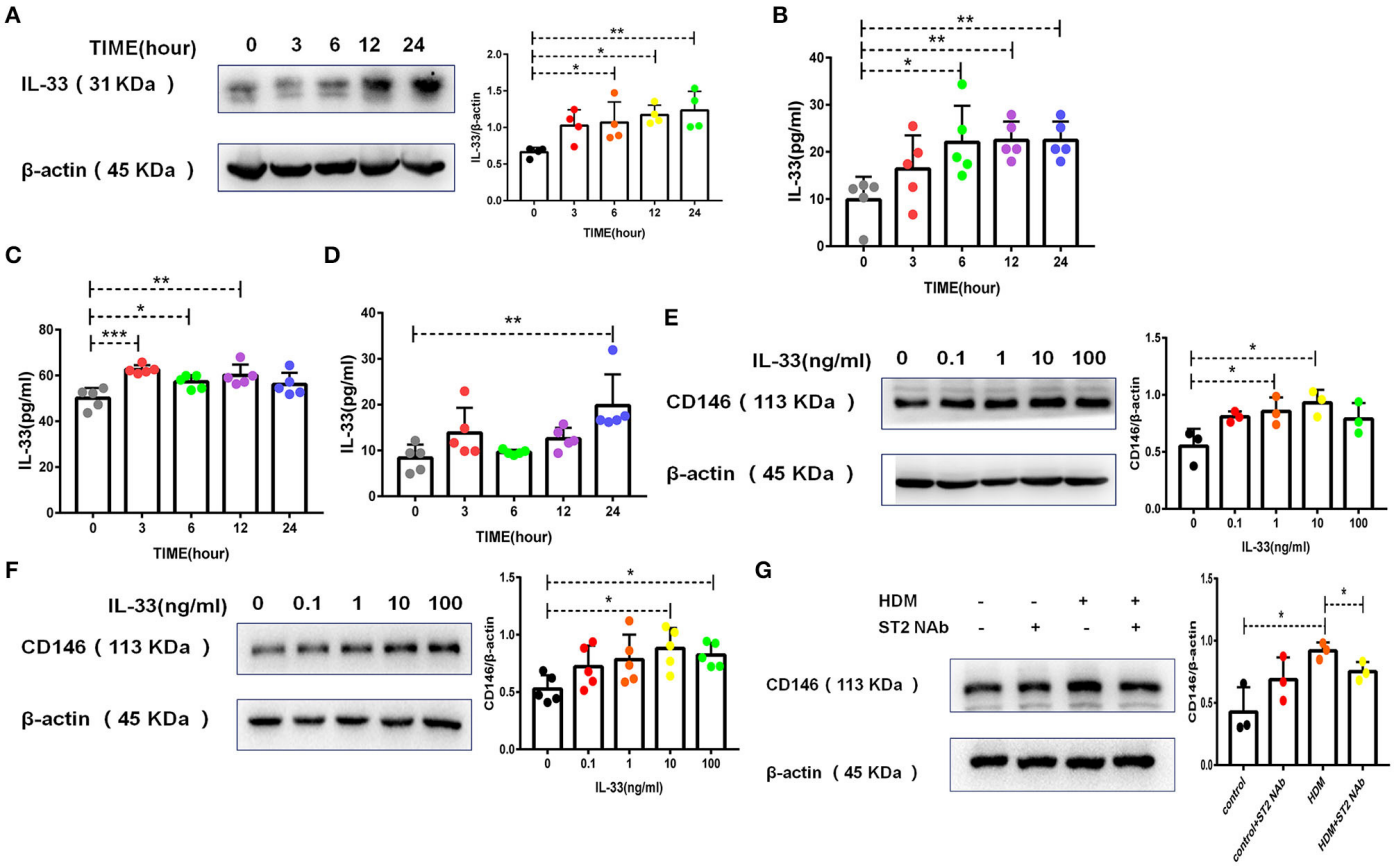
HDM promoted CD146 expression in alveolar epithelial cells via IL-33/ST2 signaling. **(A)** Western blot analysis of IL-33 expression in MLE-12 cells treated with HDM extract (100 μg/ml). **(B)** ELISA analysis of IL-33 levels in the cell culture supernatant of MLE-12 cells treated with HDM extract (100 μg/ml). **(C)** ELISA analysis of IL-33 levels in the cell lysates of MLE-12 cells treated with Derp1 (extracted HDM without LPS). **(D)** ELISA analysis of IL-33 levels in the cell culture supernatant of MLE-12 cells treated with Derp1 (extracted HDM without LPS). **(E)** Western blot analysis of CD146 expression in MLE-12 cells treated with IL-33 for 24 h. **(F)** Western blot analysis of CD146 expression in A549 cells treated with IL-33 for 24 h. **(G)** Western blot analysis of CD146 expression in MLE-12 cells treated with HDM extract (100 μg/ml) with or without an ST2-neutralizing antibody (5 μg/ml). *P < 0.05; **P < 0.01; ***P < 0.001.

Once inhaled into the respiratory tract, HDMs may directly stimulate alveolar epithelial cells. As shown in Figure 1A, HDM extract challenge increased CD146 transcripts in the mouse alveolar epithelial cell line MLE-12, which was further validated in the immunoblotting assay (Figure 1B). Primary alveolar epithelial cells purified from the lung were subjected to SPD staining (Figure 1C). Similarly, HDM extract increased CD146 expression in primary alveolar epithelial cells (Figure 1D). In agreement with a previous study that showed that IL-33 was increased in asthma (29), HDM extract increased IL-33 expression (Figure 2A) and secretion (Figure 2B) in alveolar epithelial cells. To explore whether HDM-mediated IL-33 induction was associated with the major HDM component Derp or LPS contamination, we removed endotoxin from HDM extract and treated epithelial cells with treated HDM that lacked LPS. Again, IL-33 was increased in the cell lysate (Figure 2C) or culture supernatant (Figure 2D) was increased.

To explore whether IL-33 is involved in CD146 expression, we stimulated epithelial cells with IL-33 and found that IL-33 directly promoted CD146 expression in mouse alveolar epithelial MLE-12 cells (Figure 2E) and human alveolar epithelial A549 cells (Figure 2F). The ST2-neutralizing antibody decreased CD146 expression (Figure 2G), suggesting that IL-33/ST2 was required for CD146 expression in HDM-treated epithelial cells. In summary, HDM extract increased the expression of CD146 in alveolar epithelial cells, which was mediated by IL-33 and its receptor ST2.

### CD146 Expression in Alveolar Epithelial Cells Was Dependent on p65

**Figure 3:**
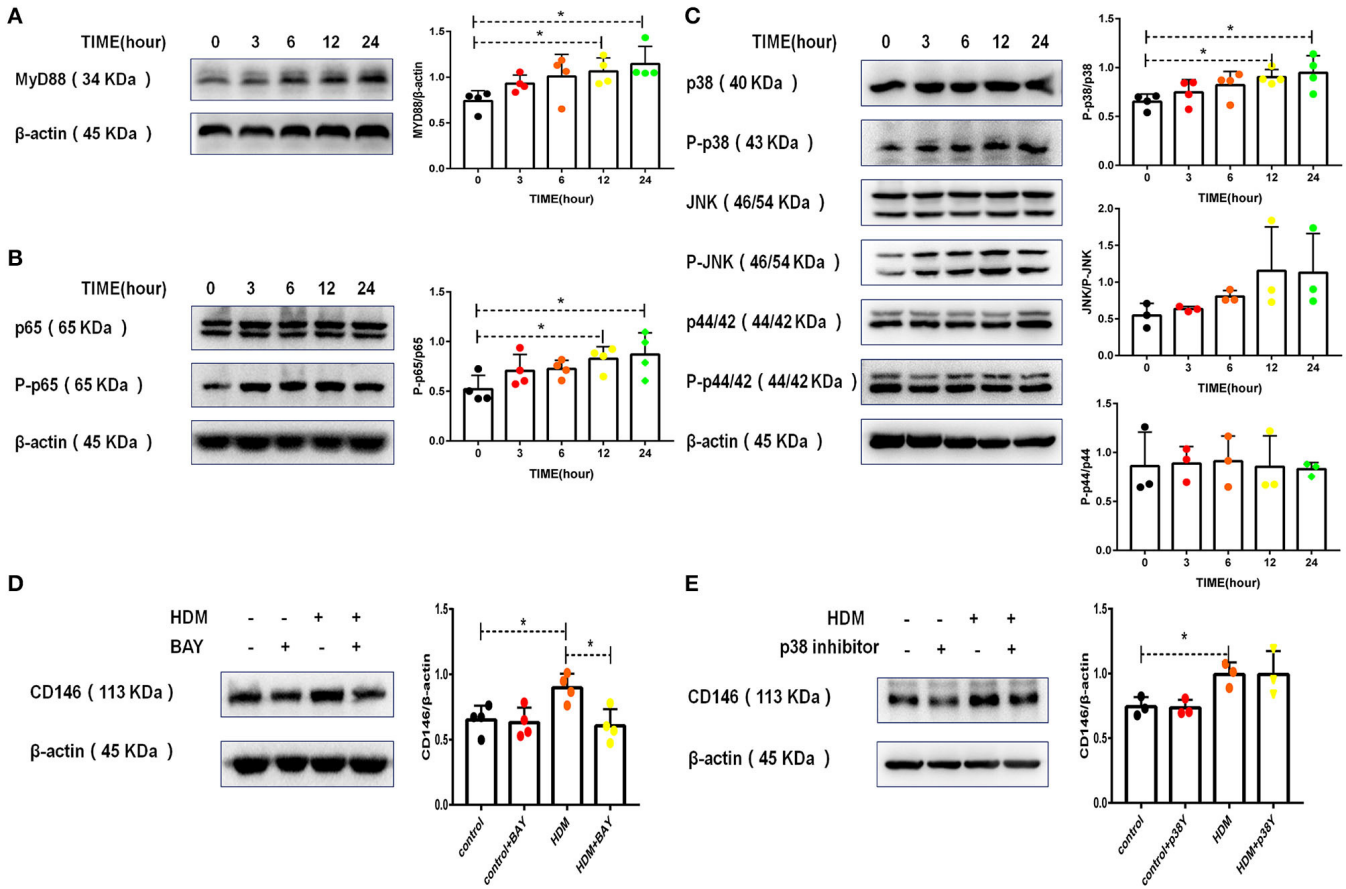
CD146 expression in alveolar epithelial cells was dependent on p65. **(A)** Western blot analysis of MyD88 expression in MLE-12 cells treated with HDM extract (100 μg/ml). **(B)** Western blot analysis of NF-κB p65 expression in MLE-12 cells treated with HDM extract (100 μg/ml). **(C)** Western blot analysis of MAPK expression in MLE-12 cells treated with HDM extract (100 μg/ml). **(D)** Western blot analysis of CD146 expression in MLE-12 cells treated with HDM extract (100 μg/ml) and a p65 inhibitor (BAY, 10 μm) for 24 h. **(E)** Western blot analysis of CD146 expression in MLE-12 cells treated with HDM extract and a p38 inhibitor (SB203580, 10 μm) for 24 h. *P < 0.05.

IL-33 binding to ST2 on epithelial cells may activate a series of downstream signaling pathways, including the MyD88, NF-κB, and MAPK pathways (30). As shown in Figure 3A, HDM extract activated MyD88 in MLE-12 cells. Similarly, HDM extract increased the phosphorylation of NF-κB p65 (Figure 3B). In the MAPK signaling pathway, p38 but not JNK, and p42 was activated in MLE-12 cells treated with HDM extract (Figure 3C). More importantly, the p65 inhibitor antagonized the HDM-induced upregulation of CD146 (Figure 3D), highlighting the importance of NF-κB in CD146 expression. In contrast with the results observed with the p65 inhibitor, the p38 inhibitor showed insignificant effects on the expression of CD146 in MLE-12 cells treated with HDM extract (Figure 3E). Therefore, CD146 in HDM-treated alveolar epithelial cells was regulated by NF-κB p65.

### HDM Promoted EMT in Alveolar Epithelial Cells via CD146

**Figure 4:**
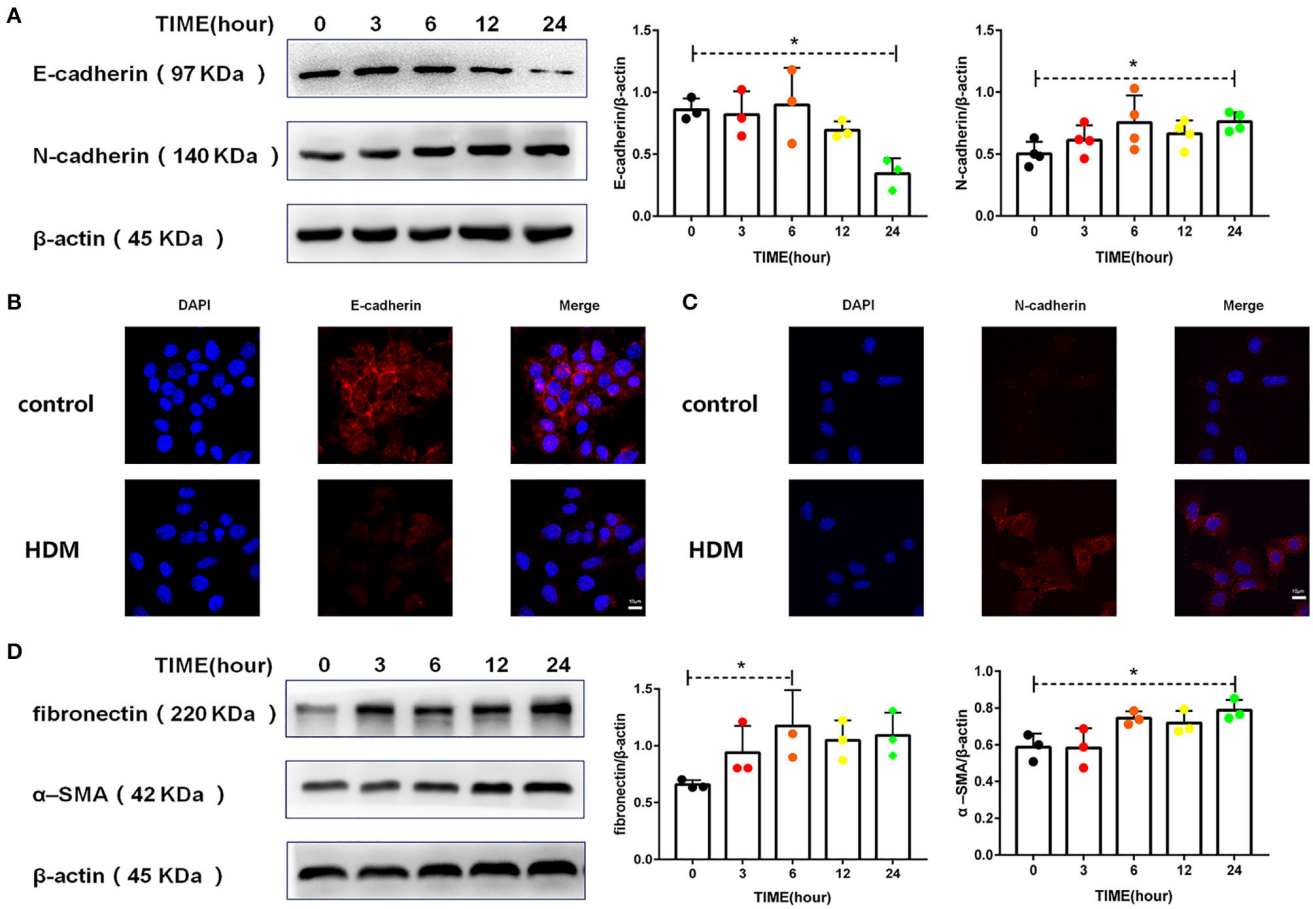
HDM promoted EMT in alveolar epithelial cells. **(A)** Western blot analysis of E-cadherin and N-cadherin expression in MLE-12 cells treated with HDM extract (100 μg/ml). **(B)** Immunofluorescence analysis of E-cadherin expression in MLE-12 cells treated with HDM extract (100 μg/ml) for 24 h. **(C)** Immunofluorescence analysis of N-cadherin expression in MLE-12 cells treated with HDM extract (100 μg/ml) for 24 h. **(D)** Western blot analysis of fibronectin and α-SMA expression in MLE-12 cells treated with HDM extract (100 μg/ml). *P < 0.05.

There is now evidence that asthma patients have more EMT than normal individuals (5, 31). The cadherin switch, which is a fundamental event in EMT, was induced in MLE-12 cells treated with HDM extract. As shown in Figures 4A–C, HDM extract decreased the expression of E-cadherin and increased N-cadherin expression. In addition, HDM extract increased fibronectin and α-SMA levels in MLE-12 cells (Figure 4D), suggesting that HDM extract promoted EMT in alveolar epithelial cells.

**Figure 5:**
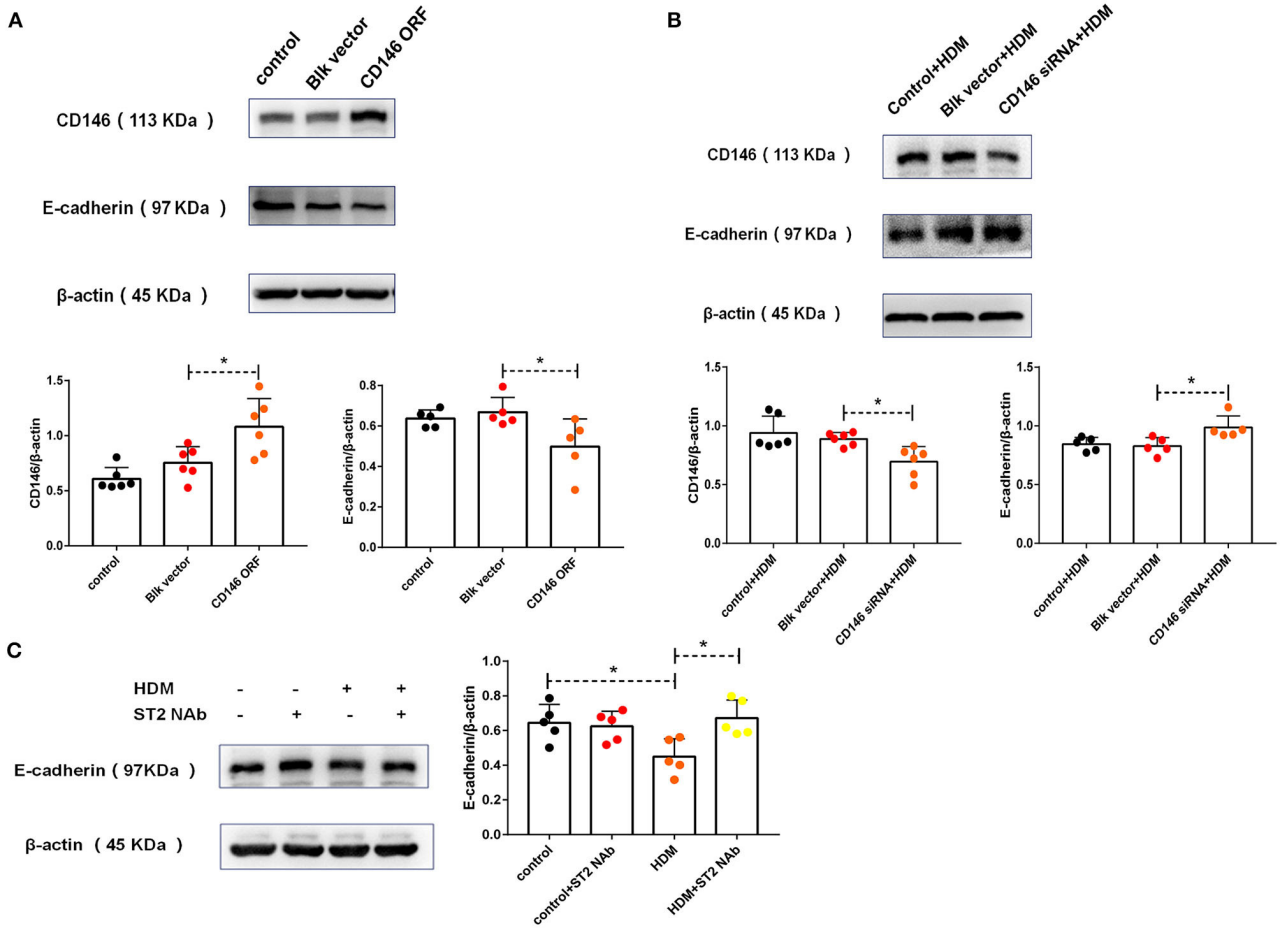
HDM promoted EMT in alveolar epithelial cells via CD146. **(A)** Western blot analysis of CD146 and E-cadherin expression in MLE-12 cells treated with a CD146 expression plasmid (CD146 open reading frame, CD146 ORF) or blank vehicle (Blk vector). **(B)** Western blot analysis of CD146 and E-cadherin expression in MLE-12 cells treated with a CD146 siRNA plasmid and HDM extract (100 μg/ml). **(C)** Western blot analysis of E-cadherin expression in MLE-12 cells treated with HDM extract (100 μg/ml) and an ST2-neutralizing antibody (5 μg/ml). *P < 0.05.

To explore the roles of CD146 in HMD-induced EMT, CD146 was either overexpressed via an expression plasmid or silenced with a siRNA plasmid in MLE-12 cells. Accompanied by CD146 elevation, E-cadherin was significantly decreased (Figure 5A). In contrast, CD146 silencing caused the increased expression of E-cadherin in epithelial cells (Figure 5B). E-cadherin expression was inversely correlated with CD146 expression, suggesting that CD146 may positively regulate EMT in alveolar epithelial cells. Moreover, the ST2-neutralizing antibody rescued E-cadherin expression in epithelial cells treated with HDM extract (Figure 5C). Considering that the ST2-neutralizing antibody decreased CD146 expression in epithelial cells treated with HDM extract (Figure 2G), we concluded that IL-33/ST2 contributed to CD146-mediated EMT in alveolar epithelial cells treated with HDM extract.

### TGF-β and SMAD3 Played Dominant Roles in EMT in Alveolar Epithelial Cells Treated With HDM Extract

**Figure 6:**
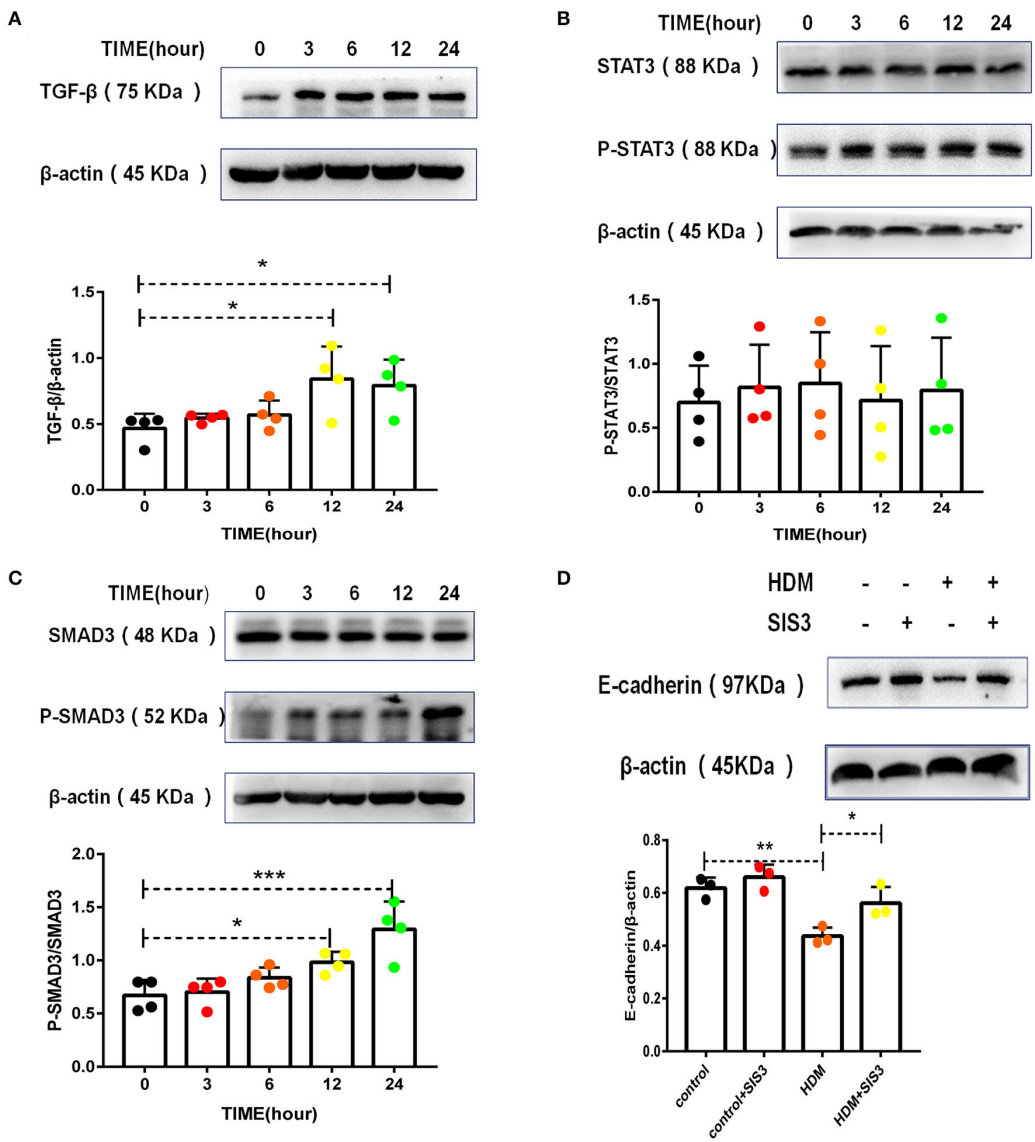
TGF-β and SMAD3 played dominant roles in HDM-treated alveolar epithelial cell EMT. **(A)** Western blot analysis of TGF-β expression in MLE-12 cells treated with HDM extract (100 μg/ml). **(B)** Western blot analysis of STAT3 expression in MLE-12 cells treated with HDM extract (100 μg/ml). **(C)** Western blot analysis of SMAD3 expression in MLE-12 cells treated with HDM extract. **(D)** Western blot analysis of E-cadherin expression in MLE-12 cells treated with HDM extract (100 μg/ml) and a SMAD3 inhibitor (SIS3, 10 μm) for 24 h. *P < 0.05; **P < 0.01;***P < 0.001.

TGF-β has been shown to be the most common EMT inducer in asthma (5, 32). Accordingly, HMD extract increased TGF-β levels in alveolar epithelial cells (Figure 6A). STAT3 and SMAD3 are downstream molecules of the TGF-β signaling pathway in the EMT process. Administration of HDM extract contributed minimally to STAT3 activation (Figure 6B) but resulted in the phosphorylation of SMAD3 (Figure 6C) in alveolar epithelial cells. More importantly, a SMAD3 inhibitor (SIS3) partially but significantly increased E-cadherin expression in MLE-12 cells treated with HDM extract (Figure 6D), suggesting that TGF-β and SMAD3 regulate EMT in HDM-treated alveolar epithelial cells.

### IL-33 Was Essential for CD146 Expression in a Mouse Model of Asthma

**Figure 7:**
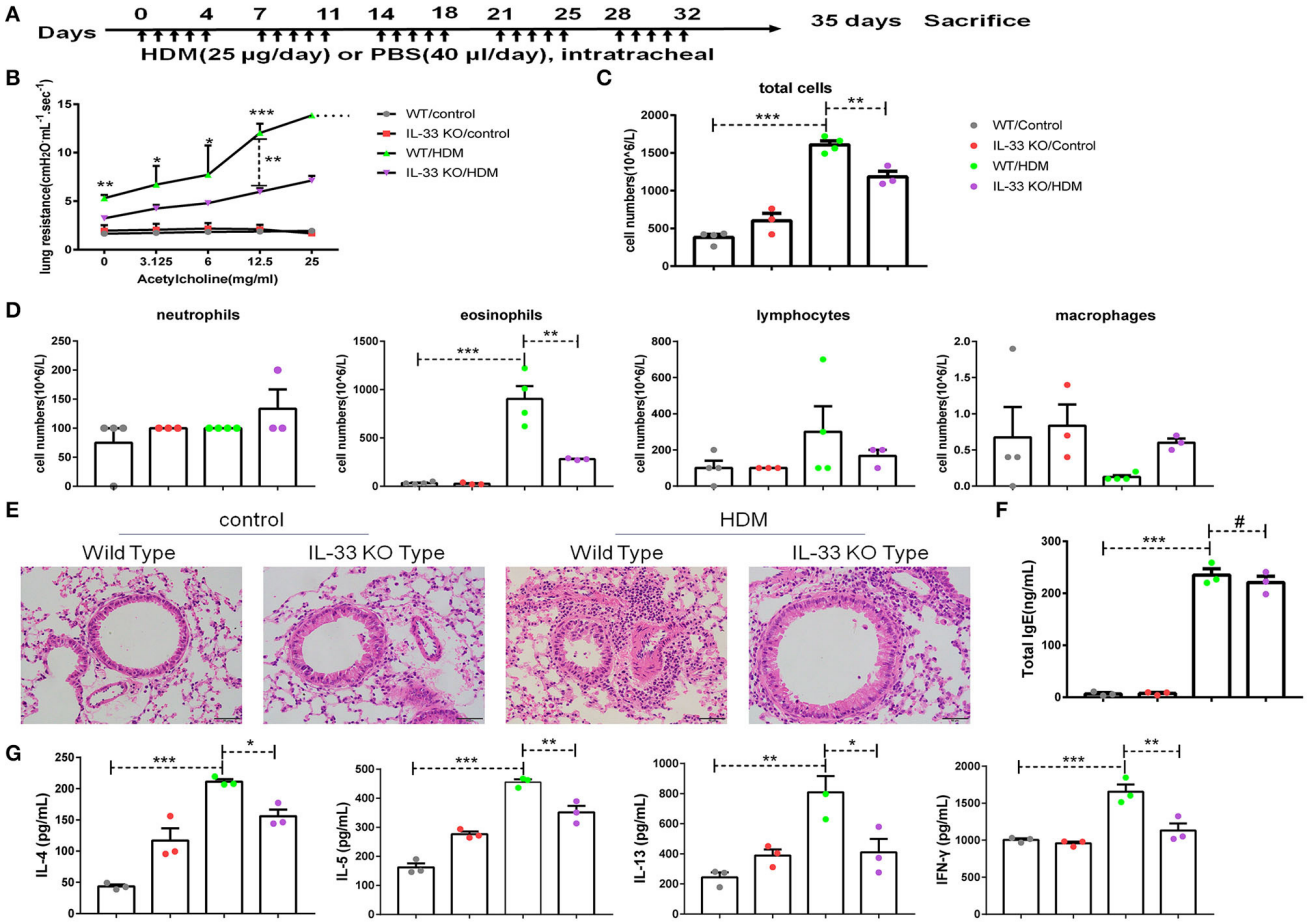
IL-33 played an important role in a mouse model of asthma. **(A)** Flow chart showing the chronic asthma model (5 days/week for 5 weeks). **(B)** Lung resistance in mice was measured with the FinePointe RC System. **(C,D)** The numbers of total cells, eosinophils, neutrophils, and lymphocytes in BALF were quantified. **(E)** Representative images of lung sections stained with H&E. **(F)** ELISA analysis of total IgE levels in sera. **(G)** ELISA analysis of IL-4, IL-5, IL-13, and IFN-γ levels in the supernatants of lung homogenates. *P < 0.05; **P < 0.01; ***P < 0.001; ^#^*P* > 0.1.

To demonstrate the significance of IL-33/ST2 in CD146 expression, we developed an asthma model in wild-type mice and IL-33 KO mice (Figure 7A). IL-33 deficiency reduced lung resistance in the murine model of asthma (Figure 7B). The number of total cells and eosinophils in BALF were decreased in the IL-33 KO mice treated with HDM extract (Figures 7C,D). Similarly, pulmonary tissue sections stained with H&E exhibited more inflammatory infiltration in WT mice than in IL-33 KO mice (Figure 7E). Total IgE in sera was significantly elevated in the HDM-treated mice; however, the IgE concentration was comparable in WT and IL-33 KO mice challenged with HDM extract (Figure 7F). The expression of type 2 cytokines, including IL-4, IL-5, and IL-13, was increased in the HDM extract-treated mice. IL-33 deficiency reduced IL-4, IL-5, IL-13, and IFN-γ levels in the lung tissue of the HMD-treated mice (Figure 7G). These results suggest that IL-33 deficiency may alleviate asthma disease severity.

**Figure 8:**
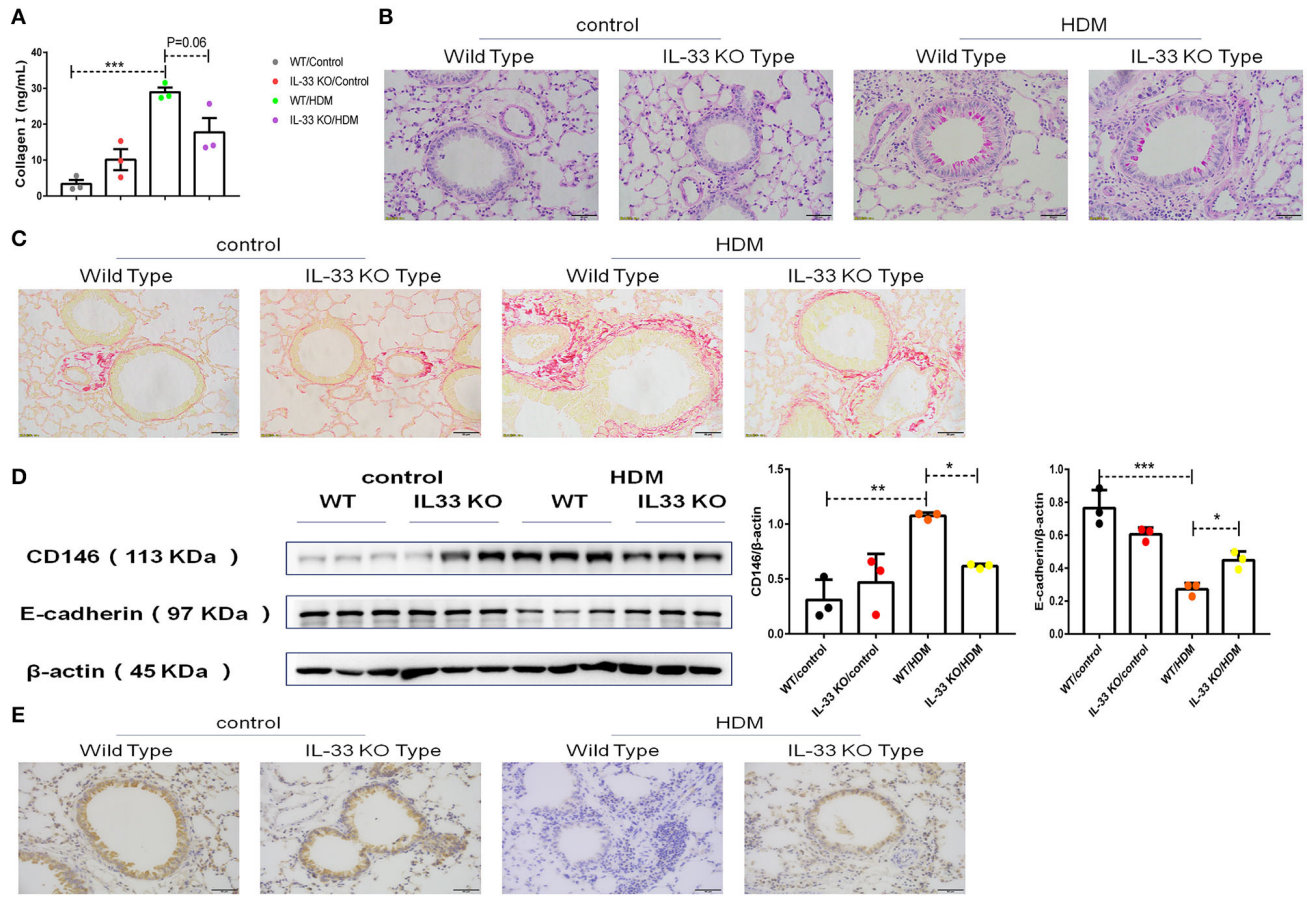
IL-33 was essential for CD146 expression and EMT in a mouse model of asthma. **(A)** ELISA analysis of collagen-I levels in the lung homogenates of mice. **(B)** Representative images of lung sections stained with PAS. **(C)** Representative images of lung sections stained with Sirius red. **(D)** Western blot analysis of CD146 and E-cadherin expression in lung tissues. **(E)** Representative images of immunohistochemical analysis of E-cadherin expression in lung tissues. *P < 0.05; **P < 0.01; ***P < 0.001.

To further explore EMT in asthma, collagen I in pulmonary tissue was quantified, and the results showed that the level of collagen I was decreased in IL-33 KO mice compared to WT mice treated with HDM extract (Figure 8A). As expected, pulmonary tissue sections stained with PAS (Figure 8B) or Sirius red (Figure 8C) revealed that collagen deposition and glycogen storage were more pronounced in WT mice than in IL-33 KO mice. Consistent with the previous *in vitro* observations, decreased CD146 (Figure 8D) and elevated E-cadherin (Figures 8D,E) levels were observed in IL-33 KO mice compared to WT type mice after HDM treatment. As observed *in vitro*, Myd88, NF-κB, and p38 may be involved in EMT in the mouse model of asthma (Supplementary Figure 1). In summary, IL-33 deficiency alleviated disease severity and decreased CD146 expression and EMT in asthma.

### CD146 Deficiency Decreased EMT in a Mouse Model of Asthma

**Figure 9:**
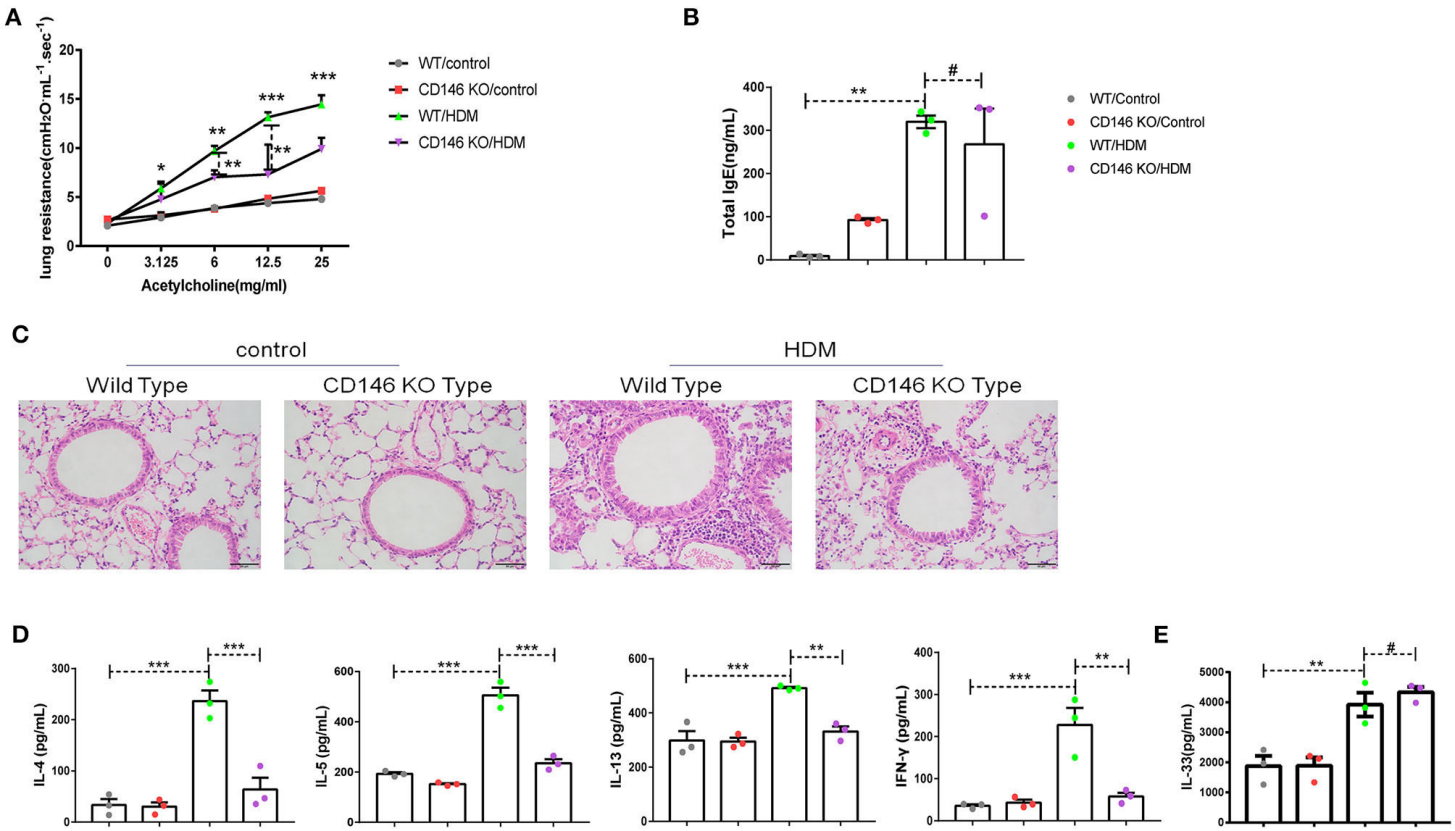
CD146 deficiency mitigated disease severity in a mouse model of asthma. **(A)** Lung resistance in mice was measured with the FinePointe RC System. **(B)** Representative images of lung sections stained with H&E. **(C)** ELISA analysis of total IgE levels in sera. **(D)** ELISA analysis of IL-4, IL-5, IL-13, and IFN-γ levels in the supernatants of lung homogenates. **(E)** ELISA analysis of IL-33 levels in the supernatants of lung homogenates. *P < 0.05; **P < 0.01; ***P < 0.001; ^#^*P* >0.05.

**Figure 10:**
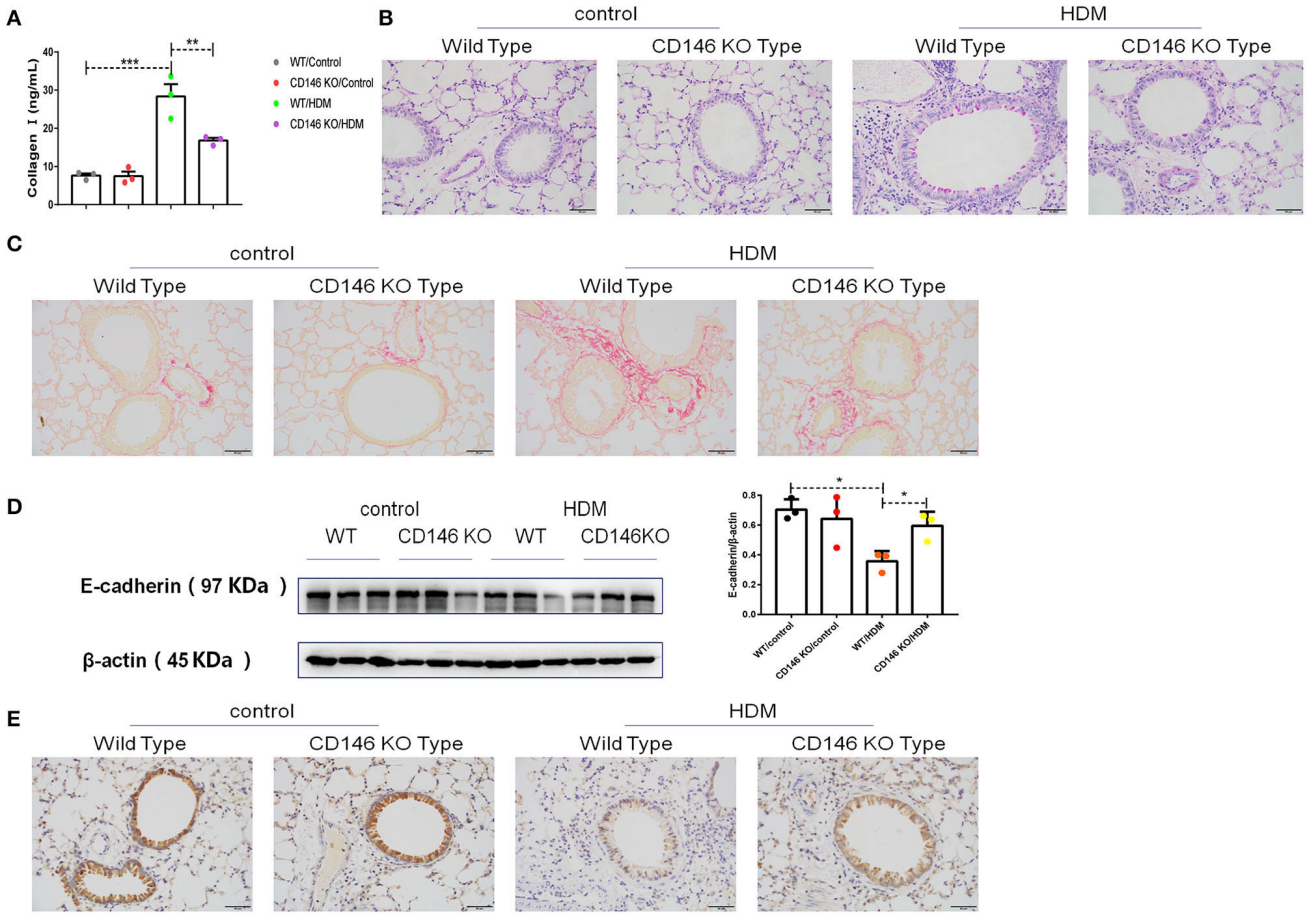
CD146 deficiency decreased EMT in a mouse model of asthma. **(A)** ELISA analysis of collagen-I levels in the supernatants of lung homogenates of WT mice and CD146 KO mice. **(B,C)** Representative images of PAS- or Sirius red-stained lung sections from WT mice and CD146 KO mice. **(D)** Western blot analysis of E-cadherin expression in lung tissues from WT mice and CD146 KO mice. **(E)** Representative images of immunohistochemical analysis of E-cadherin expression in lung tissues from WT mice and CD146 KO mice. *P < 0.05; **P < 0.01; ***P < 0.001.

To further evaluate the roles of CD146 in the asthma-associated EMT process, we established an asthma model in WT mice and CD146 KO mice. As shown in Figure 9A, lung resistance was reduced in CD146 KO mice compared to WT mice treated with HDM extract. The IgE level in the asthmatic WT mice and CD146-deficient mice was comparable (Figure 9B). In pulmonary tissues stained with H&E, the inflammatory response was decreased in the CD146 KO murine model of asthma (Figure 9C). Pulmonary cytokines, including IL-4, IL-5, IL-13, and IFN-γ, were decreased in CD146 KO mice compared to WT mice after HDM treatment (Figure 9D). Of note, IL-33 levels in asthmatic WT or CD146-deficient mice were comparable (Figure 9E). Because CD146 regulated EMT in alveolar epithelial cells, the level of collagen I was significantly decreased in the mouse model of asthma with a CD146 KO background (Figure 10A). Similarly, collagen deposition and glycogen storage in asthmatic CD146 KO mice were decreased, as evidenced by PAS (Figure 10B) and Sirius red staining (Figure 10C), respectively. Furthermore, CD146 deficiency caused an increase in E-cadherin in the asthma model (Figures 10D,E), suggesting that CD146 may orchestrate EMT in asthma.

### Soluble CD146 Was Elevated in the Plasma of Asthma Patients

**Figure 11:**
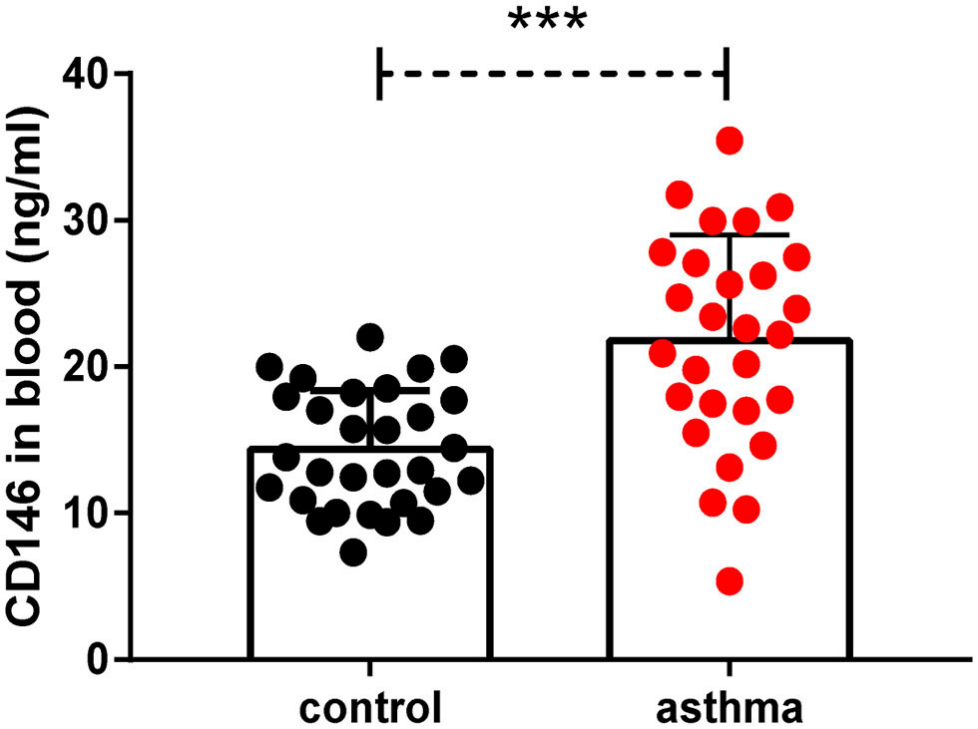
Soluble CD146 was elevated in the plasma of asthma patients. The level of soluble CD146 in the plasma of asthmatic patients and healthy people was measured by ELISA. ***P < 0.001.

We demonstrated that CD146 contributed to asthma pathogenesis in a mouse model. CD146 is not only expressed on the cell membrane but could also be released into circulation (33). To demonstrate the clinical significance of the study, we measured soluble CD146 (sCD146) levels in the plasma of asthma patients. As shown in Figure 11, the level of sCD146 was significantly increased in asthma patients compared to healthy controls. Considering that CD146 was increased in the airway epithelial cells of asthma patients (11, 12), we hypothesized that CD146 may be important in asthma.

## Discussion

In the present study, we first demonstrated that HDM extract promoted CD146 expression in alveolar epithelial cells via IL-33, and this effect was blocked with an antibody against the IL-33 receptor ST2. CD146, which was upregulated with an expression plasmid or downregulated with an siRNA plasmid, was found to play essential roles in E-cadherin expression in alveolar epithelial cells, suggesting that CD146 may mediate EMT in asthma. In a chronic asthma model in IL-33-deficient mice, CD146 expression was decreased in the pulmonary tissues, accompanied by increased E-cadherin expression, suggesting that IL-33 is essential in the CD146 expression and airway remodeling observed in asthma. Accordingly, CD146 deficiency in this chronic asthma model caused elevated E-cadherin expression, suggesting that CD146 deficiency reduced EMT in asthma. Moreover, we found that the level of soluble CD146 was increased in asthma patients. Therefore, we hypothesized that CD146 may mediate airway remodeling in chronic asthma in a manner that was dependent on the IL-33 signaling pathway.

In pulmonary epithelial cells, HDM extract stimulated CD146 expression and IL-33 production. As an alarmin molecule (34) and mucosal response amplifier (35), IL-33 binding with its receptor ST2 promoted CD146 expression. It has been demonstrated that IL-33 receptor knockout decreases the airway inflammatory response but induces the persistence of IL-5^+^ IL-13^+^ type 2 innate lymphocytes to maintain certain characteristics of asthma (36). Consistent with the above observation, we observed that IgE levels were comparable in the WT and IL-33 KO murine asthma models. In HDM-treated CD146 KO mice, the IL-33 concentration was similar to that in HDM-treated WT mice and was accompanied by comparable IgE levels in WT and CD146 KO mice treated with HDM extract. These results suggest that IL-33 was not indispensable for IgE induction in asthma.

EMT has been reported to be intricately involved in airway remodeling in asthma (37, 38). In contrast, inhibition of the EMT process may slow airway remodeling in asthma (39). The increased expression of IL-33 in airway epithelial cells is closely related to the severity of asthma (40), and IL-33 has been shown to not only exacerbate airway inflammation (41) but also promote airway remodeling in asthma (42–44). Downstream signaling molecules of ST2, including MyD88, NF-κB p65, and MAPK, are then activated. However, only NF-κB p65 was indispensable for the CD146 expression observed in alveolar epithelial cells after stimulation with HDM extract. Because CD146 dimerization may activate NF-κB p65 (45), the reciprocal regulatory mechanisms between CD146 and NF-κB p65 warrant further study.

CD146 has been shown to be expressed by diverse cell types with multiple functions (7). In mouse tracheal epithelial cells, CD146 expression was accompanied by IL-13-mediated eotaxin-3 expression, suggesting that CD146 is an enhancer of the IL-13 response (46). In human primary nasal airway epithelial cells stimulated with TLR agonists, the absence of CD146 decreased expression of the inflammatory chemokine IL-8 (47), suggesting that CD146 may amplify inflammation. Consistent with the roles of CD146 in the inflammatory response, IL-4, IL-5, IL-1, and IFN-γ levels were significantly reduced in CD146-deficient mice with chronic asthma. Moreover, CD146 was directly linked to EMT in alveolar epithelial cells, and this relationship was dependent on the TGF-β/Smad-3 signaling pathway.

CD146 has been shown to be expressed on not only epithelial cells but also other cells, including endothelial cells (48), subpopulations of T cells (49), and mesenchymal stromal cells (MSCs) (50). All of these cell types may be involved in asthma pathogenesis and tissue remodeling (51, 52). In addition to epithelial cells, the roles of other CD146^+^ cells in EMT and airway remodeling in asthma need to be elucidated in the future. Moreover, CD146 is shed from the cell membrane via MMP-3 activity (53). Elevated sCD146 levels in the plasma of asthma patients may enhance the production of vascular endothelial growth factor receptor (VEGFR) and VEGF2 (54). Therefore, we hypothesized that CD146 also regulated neovascularization, which is closely associated with EMT in asthma (55).

In summary, we expanded the role of CD146 in the EMT process from cancer metastasis to airway remodeling in asthma. We proposed that the binding of IL-33 to ST2 on HDM-stimulated airway epithelial cells promoted CD146 expression, which further amplified the inflammatory response, EMT and airway remodeling.

## Data Availability Statement

All datasets generated for this study are included in the article/Supplementary Material.

## Ethics Statement

The studies involving human participants were reviewed and approved by ethics committee of the First Affiliated Hospital of Nanjing Medical University (2017-SR-298). The patients/participants provided their written informed consent to participate in this study. The animal study was reviewed and approved by Animal Care and Use Committee of Nanjing Medical University (IRB: 1709011).

## Author Contributions

ZS, MH, and MZ designed the experiments. ZS, QM, RZ, ZC, ZW, and FH performed the experiments and analyzed the data. NJ and CW collected and characterized the clinical samples. MH and MZ conceived and supervised the project. ZS and MZ wrote the manuscript. All authors approved the final version of the manuscript.

## Conflict of Interest

The authors declare that the research was conducted in the absence of any commercial or financial relationships that could be construed as a potential conflict of interest.

## Supplementary Material

Supplementary Figure 1 Western blot analysis of MyD88, p38, and p65 expression in lung tissues. ^**^*P* < 0.01; #*P* > 0.1.
