# Supplementary figures and images for: Epithelial-Mesenchymal Transition in Asthma Airway Remodeling Is Regulated by the IL-33/CD146 Axis
Source: Front Immunol. 2020 Jul 22;11:1598. doi: 10.3389/fimmu.2020.01598 (PMC7387705; doi:10.3389/fimmu.2020.01598)

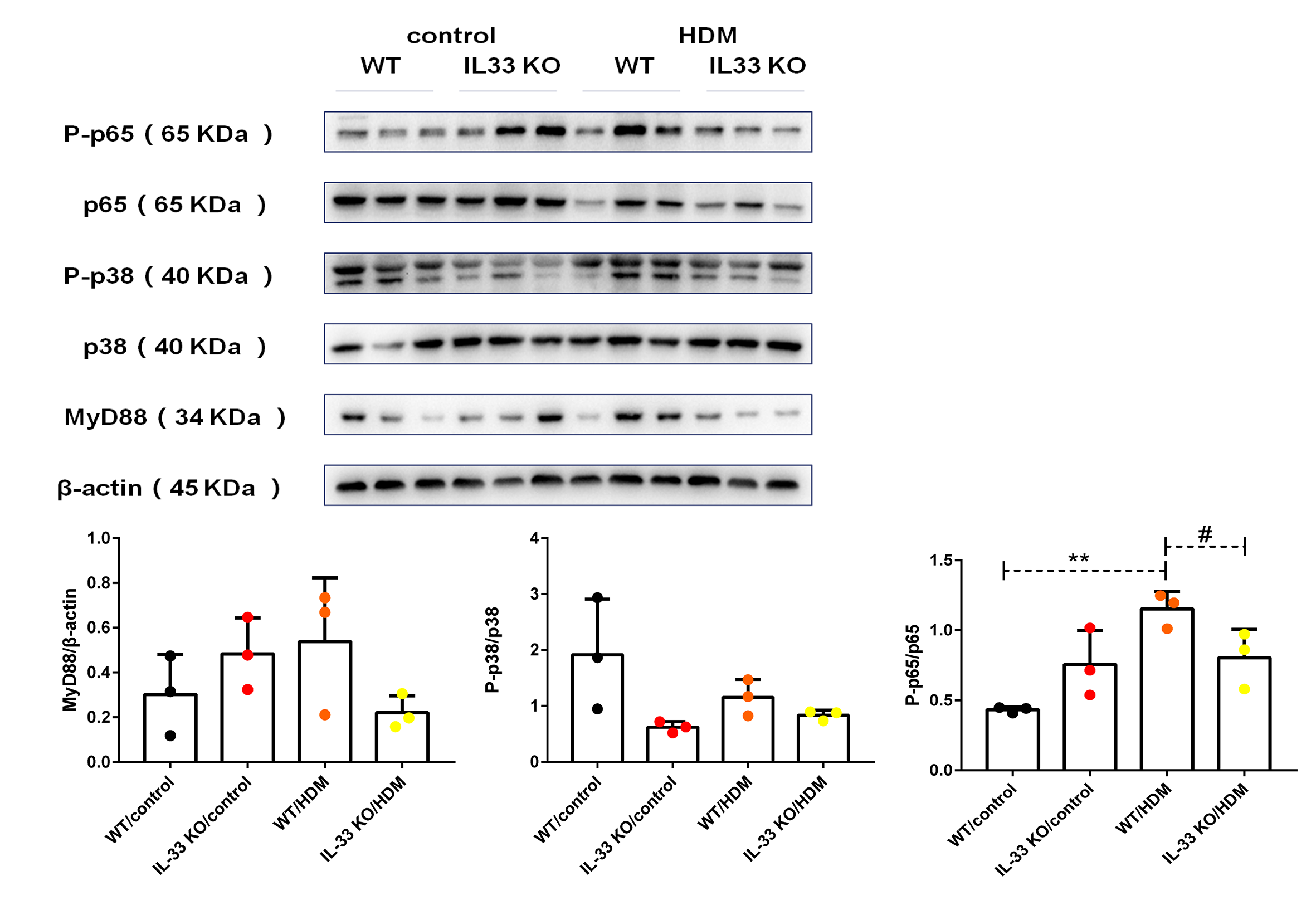

Supplement: Supplementary Figure 1 — Western blot analysis of MyD88, p38, and p65 expression in lung tissues. **P < 0.01; #P > 0.1. [file Image_1.TIF]
